# Quality of cardiovascular disease care in Ontario’s primary care practices: a cross sectional study examining differences in guideline adherence by patient sex

**DOI:** 10.1186/1471-2296-15-123

**Published:** 2014-06-18

**Authors:** Kiyuri Naicker, Clare Liddy, Jatinderpreet Singh, Monica Taljaard, William Hogg

**Affiliations:** 1 Bruyère Research Institute, C.T. Lamont Primary Health Care Research Centre, 43 Bruyère St, Annex E, Ottawa, Ontario K1N 5C8, Canada; 2 Department of Epidemiology and Community Medicine, University of Ottawa, 451 Smyth Road, Ottawa, Ontario K1H 8 M5, Canada; 3 Department of Family Medicine, University of Ottawa, 43 Bruyère St., Ottawa, Ontario K1N 5C8, Canada; 4 Ottawa Hospital Research Institute, Civic Campus, 1053 Carling Avenue, Admin Services Building, ASB 2-004, Civic Box 693, Ottawa, Ontario K1Y 4E9, Canada

**Keywords:** Sex equity, Primary care, Cardiovascular disease, Guideline adherence

## Abstract

**Background:**

Women are disproportionately affected by cardiovascular disease, often experiencing poorer outcomes following a cardiovascular event. Evidence points to inequities in processes of care as a potential contributing factor. This study sought to determine whether any sex differences exist in adherence to process of care guidelines for cardiovascular disease within primary care practices in Ontario, Canada.

**Methods:**

This is a secondary analysis of pooled cross-sectional baseline data collected through a larger quality improvement initiative known as the Improved Delivery of Cardiovascular Care (IDOCC). Chart abstraction was performed for 4,931 patients from 84 primary care practices in Eastern Ontario who had, or were at high risk of, cardiovascular disease. Measures examining adherence to guidelines associated with nine areas of cardiovascular care (coronary artery disease, peripheral vascular disease (PVD), stroke/transient ischemic attack, chronic kidney disease, diabetes, dyslipidemia, hypertension, smoking cessation, and weight management) were collected. Multivariable logistic regression analysis was performed to evaluate sex differences, adjusting for age, physician remuneration, and rurality.

**Results:**

Women were significantly less likely to have their lipid profiles taken (OR = 1.17, 95% CI 1.03-1.33), be prescribed lipid lowering medication for dyslipidemia (OR = 1.54, 95% CI 1.20-1.97), and to be prescribed ASA following stroke (OR = 1.56, 95% CI 1.39-1.75). Women with PVD were significantly less likely to be prescribed ACE inhibitors and/or angiotensin receptor blockers (OR = 1.74, 95% CI 1.25-2.41) and lipid lowering medications (OR = 1.95, 95% CI 1.46-2.62) or ASA (OR = 1.59, 95% CI 1.43-1.78). However, women were more likely to have two blood pressure measurements taken and to be referred to a dietician or weight loss program. Male patients with diabetes were less likely to be prescribed glycemic control medication (OR = 0.84, 95% CI 0.74-0.86).

**Conclusions:**

Sex disparities exist in the quality of cardiovascular care in Canadian primary care practices, which tend to favour men. Women with PVD have a particularly high risk of not receiving appropriate medications. Our findings indicate that improvements in care delivery should be made to address these issues, particularly with regard to the prescribing of recommended medications for women, and preventive measures for men.

## Background

The World Health Organization has made a specific call for greater evaluation of the impact of health care reforms on health equity within developed nations, thereby helping ensure that individuals attain their optimum level of health regardless of their ethnicity, age, gender, sexual orientation, social class or other circumstances [1]. Over the past decade, primary care has undergone significant reform within Canada, as many provinces have instituted novel physician funding approaches, team-based care models, and placed a greater emphasis on the role of primary care in chronic disease management. Despite this energetic reform, few studies have sought to examine whether patients are receiving a comparable quality of care across primary care practices, and if not, which patient-level characteristics are associated with lower quality care in order to address potential inequities.

A large body of literature suggests that women have poorer cardiovascular disease outcomes as compared to men [2]. While reasons for this disparity in cardiovascular disease outcomes are contested [3], research points to inequities in the process of care as a possible contributing factor. Some of these observed disparities may be explained by recent realizations that a misinterpretation of women’s CVD symptoms, or a lack of integration of knowledge regarding female presentations into practice, has frequently resulted in inadequate diagnoses and management in female patients [4]. One study has noted that women in primary care settings with coronary heart disease or congestive heart failure are less likely to receive cardiology consultations than men, and that consultation is associated with better processes of care, especially for women [5]. A review of patients with diabetes in Sweden reported women as having more frequent outpatient contacts, less patient satisfaction, and a lower health-related quality of life than men with diabetes [6]; however, no gender differences were found in their levels of glycemic control. Another recent study examining gender equity in primary care practices by remuneration structure found that women attending fee-for-service practices were significantly less likely to have received recommended care for chronic diseases, a difference not observed in capitation-based practices [7].

This study sought to determine whether patient sex differences exist in relation to adherence to process of care guidelines for cardiovascular disease within primary care practices in Ontario, with the goal of identifying specific gaps for improvement of equity in care delivered within the primary care system.

## Methods

### IDOCC study design

The project involves a secondary analysis of pooled cross-sectional baseline data collected through a larger quality improvement initiative known as the Improved Delivery of Cardiovascular Care (IDOCC) study [8]. IDOCC used trained facilitators to work with primary care providers within 84 primary care practices across eastern Ontario over a 24-month period, in order to help them incorporate elements of the Chronic Care Model into daily care routines for both male and female patients. Levels of adherence to CVD guidelines following this intervention were evaluated in a cluster randomized controlled trial. Baseline medical data were collected from 4,931 patients, who either have or are at high risk for developing cardiovascular disease, to study adherence rates to recommended guidelines for CVD care. The data for this study are drawn from the baseline chart abstraction, and represent patient-level guideline adherence rates prior to intervention. Further details regarding the study protocol have been published elsewhere [8]. This project has received ethical approval from the Ottawa Hospital Research Ethics Board (2007292-01H).

### Adherence to cardiovascular disease care guidelines

Data on guideline adherence were collected across nine areas of care related to CVD care: coronary artery disease, peripheral vascular disease, stroke/transient ischemic attack, chronic kidney disease, diabetes, dyslipidemia, hypertension, smoking cessation care, and weight management. The Champlain Primary Care CVD Prevention and Management Guideline (http://www.idocc.ca) was developed based on the recommendations of seven Evidence Monitoring Committees [9]. Included guidelines yielded a comprehensive list of process of care measures associated with screening, drug prescriptions, counselling and referral to external programs. Our main outcomes were dichotomous indicators of process of care measures appropriate to each of the nine areas (e.g., whether a lipid profile was taken, whether ASA or statins were prescribed, and referral to smoking cessation program), over the course of 12 months.

In addition to those patients diagnosed with one of the above conditions, a group at high-risk for cardiovascular disease was also identified. This group was identified based on the presence of at least three of the following risk factors: age (males ≥ 45, females ≥55), smoker, hypertension, and dyslipidemia [8]. Hypertension was defined as >130/80 mmHg for patients with diabetes or CKD and >140/90 mmHg for everyone else, and dyslipidemia was defined as low-density lipoprotein (LDL) >2.0 mmol/L.

### Statistical analysis

Patient- and practice-level descriptive statistics were generated for key characteristics of our sample. Baseline rates of adherence to each process of care indicator were computed for males and females. Logistic regression analysis was conducted to evaluate differences in guideline adherence for male and female patients. The method of Generalized Estimating Equations (GEE) was used to account for clustering of patients within practices. The dependent variable was a dichotomous indicator of adherence to each process of care measure. The main predictor variable was patient sex. We adjusted for practice structure, rurality, and patient age as covariates, because these have been suggested in the literature as being important predictors of quality of care [10,11]. Separate analyses were performed for patients with established disease and patients in the high-risk group. All analyses were performed using SAS 9.2 (SAS Institute Incorporated, Cary, North Carolina, USA).

## Results

Of the 4,931 patients in our sample, 48.9% were male and 51.1% were female (Table 1). The mean age was 66 years. Of these, 1,502 participants were diagnosed with CAD and 311 with PVD (Table 2). A total of 627 patients had suffered from a stroke, 69 of whom had experienced one within the previous year. A large number of patients had diabetes (2,343), chronic kidney disease (923), dyslipidemia (4,207) and/or hypertension (3,857). Over 20% (1,076) of the sample were current smokers. In addition to patients with established disease, 1,143 patients (23.2%) were identified as being at high risk for cardiovascular disease.

**Table 1:** Patient and practice characteristics

**Characteristics**	**n (%)**
Patient	4,931 (100%)
Gender (n, %)	
Male	2,411 (48.9%)
Female	2,520 (51.1%)
Age (mean, SD)	66 (11.5%)
Male	64.84 (11.3%)
Female	67.84 (12.0%)
Practice	84 (100%)
Physician Remuneration Structure	
FFS	43 (52.4%)
Capitation	27 (32.9%)
Salary- Community Health Centres	12 (14.6%)
Urban practices	69 (82.1%)

**Table 2:** Frequency of adherence to process of care guidelines in treatment of study participants

	**Percentage of patients receiving care**		
**Care indicator**	**All**	**Males**	**Females**
**All (n = 4931)**	**4931 (100%)**	**2411 (48.9%)**	**2520 (51.1%)**
Lipid profile	3968 (78.9%)	1977 (80.3%)	1991 (77.4%)
Waist circumference	518 (10.3%)	279 (11.3%)	239 (9.3%)
Dietician/weight loss program referral	931 (18.5%)	449 (18.2%)	482 (18.8%)
Smoking status recorded	4805 (95.5%)	2346 (95.4%)	2459 (95.7%)
2 Blood pressure measures	3745 (74.1%)	1778 (72.2%)	1967 (76.5%)
**High-Risk for CVD (n = 1143)**	**1143 (100%)**	**507 (44.4%)**	**636 (55.6%)**
Lipid profile	904 (79.1%)	408 (80.5%)	496 (78.0%)
Waist circumference measure	85 (7.4%)	49 (9.7%)	36 (5.7%)
Dietician/weight loss program referral	142 (12.4%)	60 (11.8%)	82 (12.9%)
Smoking status recorded	1109 (97.0%)	487 (96.0%)	622 (97.8%)
2 Blood pressure measures	811 (100%)	351 (69.2%)	460 (72.3%)
**Coronary Artery Disease (n = 1502)**	**1502 (100%)**	**931 (62.0%)**	**571 (22.4%)**
Fasting blood glucose	1197 (79.7%)	749 (80.5%)	448 (78.5%)
Medication (ACE, Angiotensin receptor blocker, beta blocker)	1339 (89.2%)	830 (89.2%)	509 (89.2%)
ASA	1156 (77.0%)	725 (77.8%)	431 (75.5%)
**Peripheral Vascular Disease (n = 311)**	**311 (100%)**	**191 (61.4%)**	**120 (38.6%)**
Fasting blood glucose	244 (78.5%)	154 (80.6%)	90 (75.0%)
ACE inhibitor and/or Angiotensin receptor blocker	182 (58.5%)	110 (57.6%)	72 (60.0%)
Lipid lowering medication	232 (74.6%)	147 (76.9%)	85 (70.8%)
ASA	238 (76.5%)	147 (77.0%)	91 (75.8%)
**Stroke (n = 627)**	**627 (100%)**	**293 (46.7%)**	**334 (53.3%)**
Fasting blood glucose	474 (76.6%)	225 (77.8%)	249 (75.5%)
ASA	493 (79.6%)	231 (79.9%)	262 (79.4%)
*If stroke within past year (n = 69)*	69 (100%)	32 (48.4%)	37 (53.6%)
Echo cardiogram	36 (52.2%)	15 (46.9%)	21 (56.8%)
Carotid doppler	46 (66.7%)	20 (62.5%)	26 (70.3%)
CT head scan	51 (73.4%)	24 (75.0%)	27 (73.0%)
EKG	38 (55.1%)	17 (53.1%)	21 (56.8%)
**Diabetes (n = 2343)**	**2343 (100%)**	**1111 (47.4%)**	**1232 (52.6%)**
Two HbA1c tests	1275 (54.4%)	622 (56.0%)	653 (53.0%)
Glycemic control medication	1882 (80.3%)	896 (80.7%)	986 (80.0%)
ACR	1327 (56.1%)	655 (58.5%)	672 (54.0%)
eGFR	1959 (83.6%)	934 (84.1%)	1025 (83.2%)
**Chronic Kidney Disease (n = 923)**	**923 (100%)**	**406 (44.0%)**	**517 (56.0%)**
ACR	488 (52.9%)	233 (57.4%)	255 (49.3%)
**Dyslipidemia**^**† **^**(n = 4207)**	**4207 (100%)**	**2124 (50.5%)**	**2083 (49.5%)**
Lipid profile	3534 (83.2%)	1799 (83.8%)	1735 (82.6%)
Lipid lowering medication	3879 (91.4%)	1993 (92.9%)	1886 (89.8%)
**Hypertension**^**‡ **^**(n = 3857)**	3857 (100%)	1827 (47.4%)	2030 (52.6%)
Two blood pressure readings	3083 (79.2%)	1407 (77.1%)	1649 (81.2%)
Anti-Hypertensive medication	3627 (94.0%)	1713 (93.8%)	1914 (94.3%)
**Smoking (n = 1076)**	**1076 (100%)**	**557 (51.8%)**	**519 (48.2%)**
Smoking cessation counselling	567 (52.7%)	294 (52.8%)	273 (52.6%)
Smoking cessation program	86 (8.0%)	46 (8.3%)	40 (7.7%)
Smoking cessation drug	249 (23.0%)	128 (23.0%)	121 (23.3%)

^**†**^Dyslipidemia defined as low-density lipoprotein (LDL) >2.0 mmol/L.

^**‡**^Hypertension defined as >130/80 mmHg for patients with diabetes or CKD; >140/90 mmHg for everyone else.

Of the 84 participating practices, approximately half (52.4%) had a fee for service remuneration structure, one third (32.9%) had a capitation-based structure, and 14.6% were salary-based community health centres (Table 1). The majority of these practices (82.1%) were located in urban areas.

Overall, women were significantly less likely to have their lipid profiles taken (OR = 1.17 [95% CI, 1.03-1.33]), be prescribed lipid lowering medication for their dyslipidemia (OR = 1.54 [95% CI, 1.20-1.97]), or to be prescribed ASA following stroke (OR = 1.56 [95% CI, 1.39-1.75]) than men (Table 3). In particular, women with PVD were significantly less likely to be prescribed ACE inhibitors and/or angiotensin receptor blockers (OR = 1.74 [95% CI, 1.25-2.41]), lipid lowering medications (OR = 1.95 [95% CI, 1.46-2.62]) or ASA (OR = 1.59 [95% CI, 1.43-1.78]). However, they were generally more likely to have two blood pressure measurements than men (OR = 0.83 [95% CI, 0.71-0.96]) and to be referred to either a dietician or weight loss program (OR = 0.87 [95% CI, 0.76-0.98]). Conversely, male patients with diabetes were less likely to be prescribed glycemic control medication than women (OR = 0.84 [95% CI, 1.43-1.78]), and hypertensive men were less likely to have two blood pressure readings taken (OR = 0.79 [95% CI, 0.66-0.94]). It is also worth noting that the odds ratios observed were higher than expected from the raw data following adjustment for age, which was a highly significant covariate across most of the models, with an observed effect in the direction opposite to that of patient sex.

**Table 3:** **Adjusted odds ratios comparing adherence to care guidelines for female versus male patients**^**†**^

**Care indicator**	**OR**	**95% CI**	**P-value**
**All** (n = 4931)			
Lipid profile not taken	**1.17**	**[1.03-1.33]**	0.01*
Waist circumference not measured	1.17	[0.93-1.47]	0.17
Dietician/weight loss program - no referral	**0.87**	**[0.76-0.98]**	0.04*
Smoking status not recorded	1.08	[0.84-1.39]	0.54
2 Blood pressure measures not taken	**0.83**	**[0.71-0.96]**	0.01*
**Coronary Artery Disease** (n = 1502)			
Fasting blood glucose not taken	1.08	[0.82-1.42]	0.56
No medication prescribed (ACE, Angiotensin receptor blocker, beta blocker)	1.07	[0.74-1.55]	0.72
ASA not prescribed	1.13	[0.86-1.49]	0.37
**Peripheral Vascular Disease** (n = 311)			
Fasting blood glucose not checked	1.00	[0.88-1.14]	0.98
ACE inhibitor and/or Angiotensin receptor blocker not prescribed	**1.74**	**[1.25-2.41]**	<0.01**
Lipid lowering medication not prescribed	**1.95**	**[1.46-2.62]**	<0.01**
ASA not prescribed	**1.59**	**[1.43-1.78]**	<0.01**
**Stroke** (n = 627)			
Fasting blood glucose not checked	1.00	[0.88-1.14]	0.98
ASA not prescribed	**1.56**	**[1.39-1.75]**	<0.01**
*If stroke within past year (n = 69)*			
Echo cardiogram not recommended	0.74	[0.29-1.91]	0.53
Carotid Doppler not recommended	0.56	[0.82-2.31]	0.42
CT head scan not recommended	1.39	[0.37-5.23]	0.63
EKG not recommended	0.59	[0.16-2.12]	0.42
**Diabetes** (n = 2343)			
Two HbA1c tests not recommended	0.93	[0.81-1.07]	0.33
Glycemic control medication not prescribed	**0.84**	**[0.74-0.96]**	<0.01**
ACR test not recommended	0.93	[0.82-1.05]	0.22
eGFR test not recommended	1.00	[0.90-1.12]	0.96
**Chronic Kidney Disease** (n = 923)			
ACR test not recommended	0.93	[0.82-1.04]	0.20
**Dyslipidemia** (n = 4207)			
Lipid profile not taken	1.09	[0.94-1.27]	0.23
Lipid lowering medication not prescribed	**1.54**	**[1.20-1.97]**	<0.01**
**Hypertension** (n = 3857)			
2 Blood pressure measured not taken	**0.79**	**[0.66-0.94]**	0.01*
Anti-Hypertensive medication not prescribed	1.09	[0.86-1.38]	0.48
**Smoking** (n = 1076)			
Smoking cessation counselling - no referral	0.94	[0.74-1.20]	0.63
Smoking cessation program - no referral	0.94	[0.61-1.47]	0.80
Smoking cessation drug - not prescribed	0.94	[0.72-1.23]	0.68

^†^Adjusted for patient age, practice fee structure and rurality.

*Significant at p = 0.05 level, **p = 0.01 level.

No statistically significant differences in delivery of care by sex were observed in patients with coronary artery disease and chronic kidney disease. Male and female patients who smoked were equally likely to receive referrals for smoking cessation counselling or programs, and to be prescribed smoking cessation medications (Table 3). In addition, no differences in adherence to overall care guidelines were observed between sexes in the group identified as high-risk for cardiovascular disease (Table 4).

**Table 4:** **Adjusted odds ratios comparing adherence to CVD care guidelines in high risk group (n = 1143)**^**†**^

**Care indicator**	**OR**	**95% CI**	**P-value**
Lipid profile not taken	1.11	[0.85 – 1.46]	0.45
Waist circumference not measured	1.39	[0.77 – 2.50]	0.28
Dietician/weight loss program – no referral	0.78	[0.69 – 1.13]	0.19
Smoking status not recorded	0.52	[0.22 – 1.24]	0.14
2 Blood pressure measures not taken	0.92	[0.69 – 1.21]	0.55

^†^Adjusted for patient age, practice fee structure and rurality.

## Discussion

The overall results of this study reveal some important sex differences in the adherence to cardiovascular disease care guidelines in primary care practices in Eastern Ontario, notably with respect to medication use and preventive measures. The main trends we observed included lower rates of lipid-related procedures and medications recommended for women; much lower rates of guideline adherence specific to peripheral vascular disease in women; unequal rates of overall medication prescription (e.g., ASA, lipid-lowering medication, and glycemic control medication) between sexes; and lower rates of screening or preventive procedures (e.g., dietician/weight loss program referral, and blood pressure measurement) in men.

While overall, women were less likely to have their lipid profiles taken, men and women with a confirmed diagnosis of dyslipidemia were equally likely to have these taken. This implies that while screening tests for dyslipidemia are less likely to be ordered for women, no differences in relation to lipid monitoring tests in those patients known to have dyslipidemia are apparent. However, within this group, women were still less likely to receive a lipid lowering medication for their condition. These findings point to two distinct gaps in lipid-related care for women in this sample - the first relating to detection, and the second relating to treatment by recommended medications. The latter finding is supported by several recent studies, one of which demonstrated that although total measured cholesterol levels were higher in women with diabetes (n = 835), women with diabetes had lower prescription rates of hypolipidaemic medications compared with men of a similar age [12]. Another study of arterial disease reports that women are significantly less likely than men to receive lipid-lowering agents than men, although no differences were observed in primary outcomes (e.g., cardiovascular death, MI or stroke) between the sexes [13]. A recent trial found that the degree of risk reduction associated with statin therapy following myocardial infarction is less in women than in men [14].

The most striking disease-specific results we observed were in relation to peripheral vascular disease. Women with this diagnosis were much less likely to receive any of the recommended medications for their condition (ASA, ACE inhibitors and/or angiotensin receptor blockers or lipid lowering medications) compared to men. These observed prescribing practices are also supported by a study in Swedish primary care facilities, which shows that men with PVD have higher odds of being prescribed a lipid-lowering therapy (OR = 1.3; 95% CI: 1.1-1.5), ACE inhibitors or beta blockers (OR = 1.3; 95% CI: 1.0-1.7), or antiplatelet therapy (OR = 1.5; 95% CI: 1.2-1.9) than women [15]. These trends are also reflected by much earlier research (circa the late 1980′s), which documented the age-adjusted likelihood of having a procedure related to PVD as being 1.7 times higher in men than women [16]. Together, these findings indicate that potentially little has changed in terms of inequities in the process of care for women with PVD in primary care settings over the last 20 years. Notably, the American Heart Association has recently issued a “call to action” for the improvement of PVD care in women, observing that research in women with this disease has lagged far behind that relating to men [17]. This is concerning given the severe consequences of untreated PVD, which include heart attack, stroke, limb disability or amputation [18]. The longer-term consequences of PVD may result in a disproportionate burden of disease in women if a lack of treatment leads to higher levels of complications and disease severity; indeed, a recent study found that after 4 years of follow-up, women with PVD have much greater mobility loss and faster functional decline than men [19], a finding which could be explained by process of care patterns observed here.

As mentioned above, these observed trends can also be attributed to a propensity to under-prescribe specific types of medications according to sex. We documented the under-prescribing of ASA, lipid lowering medications and ACE inhibitors and/or angiotensin receptor blockers to women with stroke, PVD and dyslipidemia. These findings are supported by those from a large recent study in a Finnish population, which demonstrated that younger women with a prior CVD event had a lower rate of ASA usage than men (although no gender differences were seen in the use of other preventive medication usage) [20]. In addition, a study using the EUROASPIRE III data showed that while statins and beta-blockers were equally prescribed to men and women, antiplatelet agents (e.g., ASA) were used less in women [12]. Notably, women in the EUROASPIRE III study were less likely to achieve their treatment goals [12].

Not all of the observed disparities in measures of care favoured men; indeed, men were less likely to be referred to weight loss programs or dieticians, as well as to have two blood pressure measures taken as per recommendations. The increased rates of referral to these programs for women might indicate a perception on the part of the physician that women are more likely to comply with such interventions. This is supported by findings on physician attitudes towards weight loss and gender, which found that in slightly overweight patients (BMI = 25 kg/m^2^), female patients are much more likely to be recommended to lose weight than male patients [21]. Interestingly, these researchers observed a reversal of this trend at higher BMI’s (BMI >32 kg/m^2^), with severely obese men more strongly encouraged to lose weight than similarly obese female patients [21]. Together with our findings, this indicates that although men overall may be less encouraged to lose weight, those at the highest risk for weight-related complications are still more likely than women to receive the appropriate care for this condition. A potentially concerning finding for men with diabetes in our sample was their reduced likelihood of receiving glycemic control medication as compared to women.

Interestingly, no sex differences were observed in guideline adherence for patients within the high cardiovascular disease risk group. This implies that processes of care for early detection and prevention of CVD are delivered equally regardless of sex within the practices in our sample. This finding is a positive one, especially in light of the literature regarding sex differences in screening for CVD within high-risk populations.

### Limitations

The clinicians participating in the IDOCC initiative did so voluntarily, and the data collected may therefore be subject to a degree of selection bias. Notably, participating practices may represent those who are generally higher performing and may be more motivated to adhere to quality or process of care recommendations. However, this selection bias is not expected to differ substantially with respect to male or female patients, and the internal validity of the comparisons should therefore not be affected. In addition, as with any study relying on chart audits for data collection, we were only able to capture activities which were reported in the patient file - those performed but not recorded would not have been included in our analysis. Similarly, we are unable to determine from this data whether the care indicators in question were offered to but refused by the patient, as the chart data only indicated whether they were ultimately performed.

## Conclusions

In conclusion, these findings highlight some important gaps in the quality of cardiovascular disease care in primary care practices, with implications for both women and men. We found areas of care in which no sex differences were observed, areas in which men fared worse than women, and areas in which women fared worse than men. The frequency and magnitude of these findings were larger with respect to inadequacies in care in women, and occurred in areas in which lack of appropriate care will lead to more severe outcomes such as peripheral vascular disease. Our findings indicate that improvements in care delivery should be made to address these issues, particularly with regard to the prescribing of recommended medications for women, and preventive measures for men.

## Abbreviations

ACE: Angiotensin converting enzyme; ASA: Acetylsalicylic acid; BMI: Body mass index; CI: Confidence interval; GEE: Generalized estimating equation; IDOCC: Improved delivery of cardiovascular care; OR: Odds ratio.

## Competing interests

The authors declare that they have no competing interests.

## Authors’ contributions

CL and JS originally conceived the idea for the study. All authors (WH, MT, KN, CL, and JS) contributed to the design, analysis, and interpretation. The analysis was done by KN with the assistance of all authors. All authors critically reviewed and approved the final manuscript.

## Pre-publication history

The pre-publication history for this paper can be accessed here:

http://www.biomedcentral.com/1471-2296/15/123/prepub
